# Could *Candida* Overgrowth Be Involved in the Pathophysiology of Autism?

**DOI:** 10.3390/jcm11020442

**Published:** 2022-01-15

**Authors:** Anna Herman, Andrzej Przemysław Herman

**Affiliations:** 1 Faculty of Health Sciences, Warsaw School of Engineering and Health, Bitwy Warszawskiej 20 18, 19 Street, 02-366 Warsaw, Poland; 2 Department of Genetic Engineering, The Kielanowski Institute of Animal Physiology and Nutrition, Polish Academy of Sciences, Instytucka 3 Street, 05-110 Jabłonna, Poland; a.herman@ifzz.pl

**Keywords:** autism, *Candida albicans*, candidiasis and autism, gastrointestinal, probiotics, fecal microbiota transplantation, microbiota transfer therapy

## Abstract

The purpose of this review is to summarize the current acquiredknowledge of *Candida* overgrowth in the intestine as a possible etiology of autism spectrum disorder (ASD). The influence of *Candida* sp. on the immune system, brain, and behavior of children with ASD isdescribed. The benefits of interventions such as a carbohydrates-exclusion diet, probiotic supplementation, antifungal agents, fecal microbiota transplantation (FMT), and microbiota transfer therapy (MTT) will be also discussed. Our literature query showed that the results of most studies do not fully support the hypothesis that *Candida* overgrowth is correlated with gastrointestinal (GI) problems and contributes to autism behavioral symptoms occurrence. On the one hand, it was reported that the modulation of microbiota composition in the gut may decrease *Candida* overgrowth, help reduce GI problems and autism symptoms. On the other hand, studies on humans suggesting the beneficial effects of a sugar-free diet, probiotic supplementation, FMT and MTT treatment in ASD are limited and inconclusive. Due to the increasing prevalence of ASD, studies on the etiology of this disorder are extremely needed and valuable. However, to elucidate the possible involvement of *Candida* in the pathophysiology of ASD, more reliable and well-designed research is certainly required.

## 1. Introduction

Autism spectrum disorder (ASD) is a neurodevelopmental disorder, which occurs in early childhood and persists into adulthood, and is manifested by a high level of social interaction insufficiency, language impairment, and repetitive behavior [1]. The etiology of autism has not been clearly defined yet. However, maternal infection during pregnancy [2,3]; maternal obesity and diabetes [4]; excessive early childhood vaccination [5,6]; heavy metal toxicity [7,8]; severe infection in the first 2 years of life [9]; involvement of bacteria, viruses and fungi [10,11]; long-term exposure to antibiotics in early life [12,13]; intestinal microbial dysbiosis [14,15]; gastrointestinal (GI) problems [16,17]; leaky gut syndrome [18,19]; allergies [20,21]; immune dysfunction [22,23,24]; neuroinflammation [25,26]; developmental abnormalities of the nervous system [27]; neurotransmitter imbalances (serotonin, dopamine, GABA, noradrenaline) [28]; metabolic factors deficiency [29,30]; genetic background [31,32,33]; environmental factors [34] and oxidative stress [35] may be involved in the development of autism. Moreover, the estimated prevalence of ASD is higher in males than in females (ratio 3:1) [36]. The higher autistic phenotypes in males compared with females could be attributed to the protective effect of estrogen, the higher diversity and predominance of probiotics in females, the lower liability of females to develop leaky gut, neuroinflammation, and excitotoxicity [37]. Children with ASD are very selective eaters (“picky eaters”), and most of them show aversions to specific food colors, textures, smells, or other foods’ characteristics [38]. Indeed, children with ASD are at increased risk of a broad spectrum of concomitant medical issues; the most prevalent are sleeping problems, epilepsy, immune dysregulation, disruption of gut microbial balance (dysbiosis), and GI disturbances [39,40]. Dysbiosis refers to an imbalance in the microbial community of the human body. In this case, pathogenic bacteria can outnumber the beneficial ones, leading to complicated disorders in the host GI tract [41]; therefore, small intestinal bacterial overgrowth (SIBO) is one of the consequences [42]. Diet-derived carbohydrates that are not fully digested in the upper gut are metabolized by bacteria in the human large intestine. These non-digestible carbohydrates influence microbial fermentation and total bacterial number in the colon [43]. High growth rates of *Clostridium histolyticum*, *Clostridium perfringens*, and *Sutterella*; a high ratio of *Escherichia/Shigella*; and a low ratio of *Bacteroidetes/Firmicutes* were generally related to GI problems [44,45,46], while the relative abundance of *Desulfovibrio*, *Clostridium* spp., and *Bacteroides vulgatus* were associated with behavior disorders [47,48,49]. In turn, the studies on gut mycobiome are still in their infancy; numerous sources reported its potential role in host homeostasis and disease development [50]. The actual role of gut fungal microbiota in ASD children has not been clearly elucidated and more studies are needed [51,52]. In this paper, the current knowledge acquired on *Candida* overgrowth in the intestine of children with ASD is summarized based on several electronic databases and hand-searched references. Moreover, the yeast influence on the immune system, brain, and behavior of children with ASD is also described. The benefits of interventions such as a carbohydrates exclusion diet, probiotic supplementation, antifungal agents, fecal microbiota transplantation (FMT), and microbiota transfer therapy (MTT) are also discussed.

## 2. Literature Search Strategy

The Scopus, PubMed, and Google Scholar databases were searched for articles. Search terms included “autism and *Candida*”, “ASD and *Candida*”, “autism and candidiasis” and “Candida metabolites and autism”. References from reviews concerning *Candida* sp. in ASD were searched for additional articles and case reports. A manual search was also conducted based on citations in the published literature.

### 2.1. Inclusion and Exclusion Criteria

Selection criteria excluded articles that examined bacterial gut microbiota in ASD, animal studies, and review articles. Additionally, publications in languages other than English were excluded.

### 2.2. Study Selection

**Figure 1:**
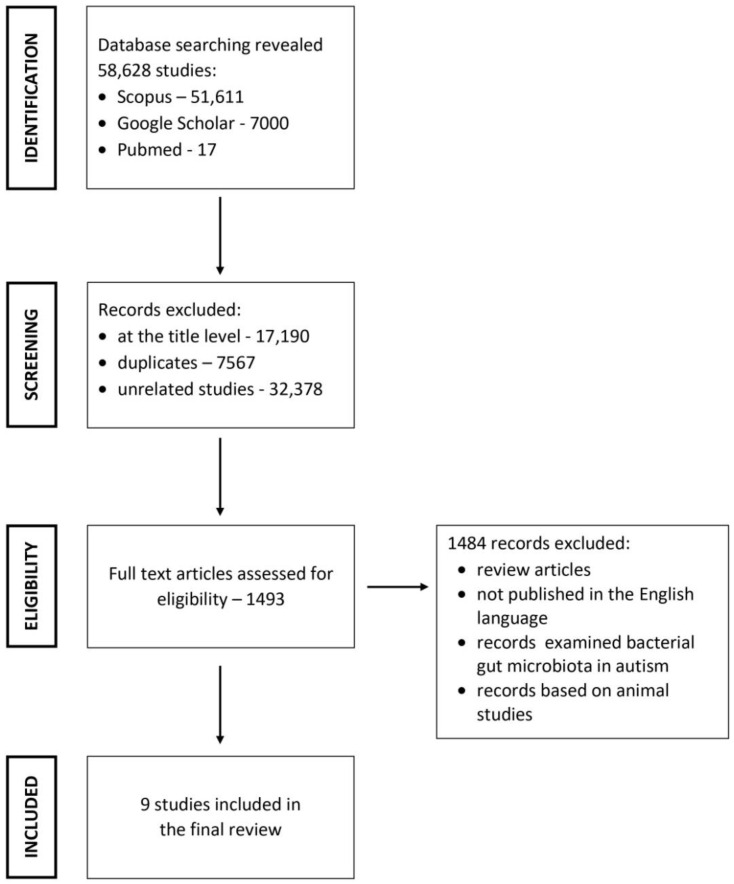
Search strategy used to identify relevant articles.

Overall, 58,628 articles were found via the search of the databases. Of these, 57,135 articles were excluded at the title level, as well as duplicates and unrelated articles. Further, 1484 articles were excluded for not meeting the inclusion criteria. Finally, nine articles were used for the review (Figure 1).

## 3. Candida and Their Metabolites Isolated from Stool, Urine, andBlood Samples from Children with ASD

The detection of clinically significant intestinal fungi growth is challenging. The early diagnosis of fungi infection remains problematic due to the poor sensitivity and specificity of current diagnostic modalities. Advances in sequencing technologies hold promise in addressing these shortcomings and for improved fungi detection and identification. *Candida albicans* germ tube antibody assay, mannan-Ag and mannan-Ab PCR, histopathology, culture, serum beta-glucan contrasted with methylene blue are successfully used [53]. The most popular tests used to diagnose *Candida* overgrowth in the gut of children with ASD (stool, urine, blood) are based on culture method, microscopic examination with staining, determination of fungi metabolites or immunoglobulins, etc., but none of these tests is completely reliable; therefore, the correct detection of the infection of candidiasis in ASD children is difficult [54].

### 3.1. Candida Isolated from Stool Samples from Children with ASD

Some studies showed that stools observed in ASD children, in comparison with those from healthy children, have different gut fungal communities [52,55]. Many studies showed that *Candida* sp. was isolated more frequently from stools from autistic patients than healthy ones (Table 1).

The genus *Candida* was identified up to twice as frequently in ASD children than in the control population [46]. However, Kantarcioglu et al. [61] reported the presence of *Candida* sp. in a large percentage of ASD patients (81.4%) compared to controls (19.6%). Emam et al. [63] also showed an increased rate of infection by yeast in the autisticgroup (81.9%) versus the control group (28%). Moreover, there was a significant relationship between autistic children and the heavy growth of *C. albicans* in stool cultures [63]. Similarly, El-Shouny et al. [64] found a heavy growth of yeast in the autistic group in comparison with the control group. Iovene et al. [59] also identified aggressive forms (pseudohyphae) of *Candida* spp. in the stools from 57% ASD children and not in age-matched healthy controls. The overgrowth of *Candida* sp. may lead to the morphological transition from budding yeast to hyphae, as well as biofilm formation; this is a key determinant in the *C. albicans* pathogenesis [65,66]. The ability of *C. albicans* to attach and disseminate from the GI tract is associated with its capacity to undergo a morphological transition from yeast to hyphae, which allows the organism to attach, invade and perpetuate the disease [67,68].

Unfortunately, although the abundance of the fungal genus *Candida* sp. was greater in autistic children than neurotypical subjects, the statistical differences in most studies were not significant [46,55,57,58,60,62] or researchers did not provide this data [56,61]. Furthermore, the methodology and degree of detection of *Candida* sp. in the stool from children with ASD affects results and statistical significance. Some literature data show that yeast were more commonly observed microscopically and only rarely observed by culture in the autistic or control groups, but the difference between these two groups was also not significant [60].

The most commonly isolated *Candida* sp. from stool samples of children with diagnosed or suspected ASD were: *C.* *albicans* (57.4%), fluconazole-resistant *C. krusei* (19.8%) and *C. glabrata* (14.8%) [61]. Moreover, *C. albicans* was the most prevalent species in both groups, while *C. krusei* and *C. glabrata* were only isolated in ASD samples [61]. Additionally, Iovene et al. [59] showed that *C. albicans* was the most frequently isolated (16 times/27 total cases) yeast from ASD patients. In turn, Ahmed et al. [57] showed that *C. glabrata* (43.1%) was the most commonly isolated from ASD patients, followed by *C. parapsilosis*(19.6%), *C. tropicalis* (17.7%), *C. albicans* (9.8%), and *C. krusei* (9.8%). El-shouny et al. [64] reported that *C. krusei* was the most commonly isolated, while Colombo et al. [69], stated that the nonalbicans *Candida* sp., such as *C. krusei*, *C. tropicalis*, were observed in their ASD cases.

Some studies attempted to link the presence of *Candida* sp. to GI dysfunction and the severity of autism symptoms (Table 1). The strong correlation of *Candida* sp. with GI symptoms and autism severity indicates that children with more severe autism are likely to have more severe GI symptoms and vice versa [46,60]. However, most of the studies showed that increased colonization with *Candida* sp. did not affect the severity of symptoms in ASD children and was not correlated with GI symptoms [55,57,58,59,62]. Moreover, the latest data showed that mycobiome dysbiosis was more pronounced in neurodevelopmental disorders, such as ASD, than in GI disorders [70]. Interestingly, probiotic intake, diet, and antibiotic exposure had a greater effect on fungal abundances than bacterial abundances, suggesting that the presence of strong interactions between dysbiosis caused by a disruption in the microbiota homeostasis and neurodevelopmental disorders, while GI disorders seemed to be associated rather with an imbalance in the bacterial community.

### 3.2. Candida Metabolites Isolated from Urine Samplesfrom Children with ASD

Some studies showed that fungi growing in the gut produced many metabolites, which affect the behavior of ASD children [52,55]. *Candida* sp. growing in the gut release many metabolites, such as arabinose [56,71], D-arabinitol [72,73], and tartaric acid [71], which are thought to contribute to autistic behaviors. Unfortunately, the association of urinary arabinose as a biomarker of intestinal yeast overgrowth is questionable. This simple sugar (aldose) is a substrate from dietary carbohydrates that, under the anaerobic conditions in the human intestinal tract, are reduced (consumed) by rapidly growing yeast to arabinitol (five-carbon sugar alcohol). This is the biochemical rationale that arabinitol, but not arabinose, is characteristic of yeast growth [74,75,76]. Furthermore, it was shown that arabinose had a little or marginal impact on *C. albicans* growth [77], and arabinose was never reported to be a metabolic product of any strain of yeast or fungus [78]. Moreover, some investigators report the ratio of D- to L-arabinitol as a biomarker of yeast overgrowth [72], but the enantiomer, L-arabinitol, is not produced by yeast but comes from the diet [74]. However, with proper calibration procedures, the measurement of D-arabinitol concentrations without the calculation of the D/L ratio is at least as sensitive as the ratio method for the detection of yeast overgrowth [74]. It was also shown that elevated levels of D-arabinitol in urine are a positive indication of *Candida* overgrowth, even if invasive candidiasis is not present [74]. Similarly, tartaric acid can be a substrate for the growth of fungi [79,80] but no literature data showed that any type of yeast or fungus could produce tartaric acid as a metabolite. To summarize, significant amounts of arabinose or tartaric acid found in urine do not confirm evidence of yeast overgrowth.

### 3.3. Candida Isolated from Blood Samples from Children with ASD

Some studies confirm the presence of filamentous fungi on blood culture or increased levels of immunoglobulins (Ig)s that target fungal antigen in the blood of ASD children. Markova et al. [81] observed cell-wall-deficient variants from the life-cycle of filamentous fungi of *Candida parapsilosis*, *Cryptococcus albidus* and *Rhodotorulamucilaginosa*, as well increased IgG, IgM, and IgA proving the presence of *Aspergillus fumigatus* in the blood of almost all autistic children in the study. The anti-Candida IgG was found in about half of the ASD children with GI dysfunction [82]. This suggests that dysbiosis could occur even with the absence of GI symptoms. Moreover, the production of IgG antibodies indicates the current or previous overgrowth of *Candida* sp. [83]. Therefore, the test for anti-Candida IgG does not clearly explain whether *Candida* sp. is present in the gut of the children with ASD at this moment, and it is not known if the treatment with antifungal drugs should be initiated. Additionally, blood cultures were assumed to be positive for *Candida* sp. in only 24 to 60% of cases [84].

## 4. The Influence *Candida* Overgrowth on the Immune System, Brain, and Behavior of ASD Children—Mechanisms of Action

In the 1980s some researchers suggested that yeasts are playing a significant role in behavior and learning problems in ASD children [85,86]. It was noted that excessive and long-term exposure to antibiotics (e.g., middle ear infection) led to gut dysbiosis and caused the overgrowth of the yeast *C. albicans* in the intestinal tract. *Candida* released many toxins into the bloodstream, which disrupted the immune system and couldinfluence the functioning of the brain, contributing to autistic behaviors. However, not all children with ASD have an overgrowth of *Candida* sp. in the gut; therefore, it is difficult to conclude that *Candida* causes autism.

### 4.1. The Dysregulation of the Immune System

Many autism susceptibility genes are localized in the immune system and are related to immune/infection pathways. They are enriched in the host–pathogen interactions of microbes (bacteria, viruses, and fungi) and to the genes regulated by bacterial toxins, mycotoxins, and Toll-like receptor ligands [87]. *C. albicans* can cause lesion formation in the gut by degrading the protective mucin layer through the action of mucolytic enzymes [88]. Simultaneously, some fatty acid metabolites secreted by gut bacterial flora modulate *C. albicans* germination [89] and hyphal growth through the target of the rapamycin (TOR) signaling pathway [90], which leads to adverse effects on the host via proinflammatory cytokines secretion [91]. *Candida* sp. colonization in the intestinal tract delays healing of inflammatory lesions [92]. These effects may create a vicious cycle where low-level inflammation promotes fungal colonization and fungal colonization promotes further inflammation. The cell wall of commensal fungi, such as *S. cerevisiae* and *C. albicans*, contains various ligands (e.g., β-glucans and chitin) for the receptors of innate immune cells [93]. The most important pathogen recognition receptors are C-type lectin receptors such as Dectin-1 and Dectin-2, mainly expressed in dendritic cells and macrophages to recognize commensal fungi [94]. After the ligand is linked to receptors, nuclear factor-kappa B (NF-κB) and mitogen-activated protein kinase (MAPKs) are activated via the CARD9-BCL10-MALT1 complex, which modulates pro-inflammatory cytokines and reactive oxygen species (ROS) production, finally restricting the growth of fungi [94]. It was shown that fungi such as *C. albicans*, *S. cerevisiae*, and *A. fumigatus* can modulate cytokine expression, particularly IL-6 [95]. The GI *Candida* colonization is associated with elevated levels of the pro-inflammatory cytokine IL-17 and enhanced inflammation [96]. It was also found that β-glucan derived from *C. albicans* can stimulate canonical inflammasomes of innate immune cells, leading to the cleavage of proinflammatory cytokines, especially IL-1β and IL-18 [97]. Moreover, it was shown that changes in IL-1β/IL-10 ratios and monocyte cytokine profiles under β-glucan-stimulated cultures indicated that changes in innate immune responses were not limited to Toll-like receptor (TLR) pathways [24]. It was also observed that autistic children with GI disease had myeloperoxidase (MPO) deficiency resulting in the exaggeration of an inflammatory response [98]. The production of MPO is the main way for neutrophils to be involved in antifungal defense, and so its deficiency may be associated with the increased fungal infection seen in many of these children. In vitro studies showed that *C. albicans*, *C. krusei*, *C. stellatoidea,* and *C. tropicalis* cannot be killed by MPO-deficient polymorphonuclear leucocytes [99]. This leads to the conclusion that killing fungi may be difficult, depending on the severity of the MPO deficiency; however, bacterial killing may not necessarily be a problem for patients with MPO deficiency [100]. On the contrary, the latest research found that serum MPO activity was significantly higher in ASD cases than in the control subjects [101]. Neutrophil levels and neutrophil/lymphocyte ratio (NLR) were also higher in ASD groups compared with healthy children [102]. Moreover, NLR significantly correlated with social interaction problems in ASD children. These findings implicated MPO as an important therapeutic target in the treatment of inflammatory conditions [103]. Therefore, curing inflammation could improve the difficult behavior and social interactions in children with ASD.

Some fungi produce toxins that regulate host immune responses. *Aspergillus fumigatus* releases gliotoxin to suppress the immune system. Gliotoxin is an inhibitor of T-cell activation and macrophage phagocytosis and induces apoptosis in monocytes and monocyte-derived dendritic cells [104,105]. *Aspergillus*-induced immune suppression in children can lead to secondary polymicrobial invasion of opportunistic bacteria and other fungal species, such as *C. parapsilosis* or *Cryptococcus albidus*. Additionally, isolates of *C. albicans* produce gliotoxins [106], immunotoxins [107,108], candidalysin [109,110], and farnesol [111] to regulate host immune responses. Moreover, candidalysin utilizes NLRP3 inflammasome-dependent cytolysis to evade phagocytic clearance [112,113], while farnesol acts as a vital virulence factor to impair the ability of immature DCs (iDC) to induce T cell differentiation and the expression of pro-inflammatory cytokines, therebyaffecting pro-inflammatory and Th1 responses [114]. Notably, fungi also secrete prostaglandins (PGs) or convert exogenous arachidonic acid into PGs [115,116] to affect the functions of phagocytes, which contribute to the continuous colonization of *C. albicans* [117]. Therefore, scientists suggest that *C. albicans* overgrowth in intestines causes higher toxin levels which are thought to contribute to autistic behaviors [118].

### 4.2. The Influence on Brain and Behavior in ASD Children

Some research suggested that *Candida* sp. are cancausenot only severe, long-term disruptions of the immune system but also attack the brain [63]. *Candida* has evolved the capacity to cross the blood–brain barrier and adhere to brain tissue [119,120]. MRI imaging using the extracellular vascular contrast agent Gd-DTPA demonstrated that the integrity of the blood–brain barrier is lost during disseminated candidiasis and both yeast and filamentous forms of the pathogen were found in the meninges and brain parenchyma in mice [121]. Moreover, it was shown that intra-amniotic exposure to *C. albicans* (10^7^ colony-forming units) caused severe systemic inflammatory and neuroinflammatory responses, illustrated by a robust increase in plasma IL-6 concentrations and concomitant white matter injury in the fetal ovine brain [122]. The cerebrospinal fluid cultures were positive for *C. albicans* in the majority of the 3-day, *C. albicans*-exposed animals, whereas no positive cultures were present in the 5-day, *C. albicans*-exposed and fluconazole-treated animals. Although *C. albicans* was not detected in the brain parenchyma, a neuroinflammatory response in the hippocampus and white matter was seen and was characterized by increased microglial and astrocyte activation. These neuroinflammatory changes were accompanied by structural white matter injury. Unfortunately, intra-amniotic fluconazole reduced fetal mortality but did not attenuate neuroinflammation and white matter injury. Immune system alterations in ASD may be implicated in the severity of behavioral impairment and other developmental outcomes. The study analyzing cytokines concentration in human blood samples showed that the concentration of IL-1β was most significantly increased in ASD patients compared with the healthy controls [123]. The studies on animal models showed that IL-1β may be responsible for the induction of some disorders which commonly accompany ASD, including reduced melatonin secretion [124] and cognitive dysfunction [125]. It is well known that *C. albicans* infection influences the secretion of IL-1 family cytokines, which are associated with acute and chronic inflammation and are essential for the innate response to infection, particularly stimulating IL-1α/β and IL-33 synthesis [126]. This may partly explain why *Candida* infections can cause or worsen the disorders associated with ASD.

Reichelt and Knivsberg [127] hypothesized that the overgrowth of *C. albicans* can be correlated with autism because yeast cells produce ammonia (NH_3_), which in combination with propionic acid, presents in the GI tract and canbe converted to β-alanine (C_3_H_9_NO_2_). β-alanine is structurally similar to the inhibitory neurotransmitter gamma-aminobutyric acid (GABA, C_4_H_9_NO_2_), which is present in higher quantities in autistic patients. The final structure for β-alanine is almost identical to GABA, except for an additional carbon atom present in GABA. The β-alanine can cross the blood–brain barrier and canbe used in the brain as a partial antagonist, blocking the receptor sites for GABA, thus facilitating the production of more GABA to achieve equilibrium [118]. An excess of GABA [128] and reduced GABA_A_ receptors in brain regions [129] was proposed as a possible contributor to autism. Unfortunately, confirmation of this hypothesis through a biochemical analysis of the reaction between propionic acid and ammonia in the context of the human body, as well as further research in terms of the dependency of β-alanine and GABA in the brain patients with autism, should be conducted.

Researchers report that *Candida* sp. may increaseintestinal production of serotonin (5-hydroxytryptamine, 5-HT) at the expense of a lower synthesis in the brain (due to consumption of its precursor tryptophan), leading to hyperserotoninemia and behavioral outcomes in ASD children [15]. Elevated whole-blood serotonin or hyperserotonemia were present in more than 25% of ASD children [130]. On the contrary, plasma 5-HT levels in autistic mothers and their ASD children were significantly lower than in mothers of normal children and differed between autistic children and their fathers and siblings [131]. Low maternal plasma 5-HT may be a risk factor for autism through effects on fetal brain development [131], and at least affect social and stereotyped behavior, sleep problems, aggression, and anxiety in ASD children [132]. Moreover, it is known that the altered metabolism of the serotonin precursor, tryptophan, occurs in autism and epilepsy, a disease very often diagnosed in children with ASD [133].

## 5. Treatment

Some interventions, i.e., diet, probiotic supplementation, antifungal agents, FMT, and MTT could alter the gut microbiota and improve behavioral symptoms among ASD patients [134,135].

### 5.1. Diet Intervention against Candida Overgrowth

Children with autism are picky eaters, and their diet is usually limited to a very narrow range of foods depending on their type, texture, or appearance [136]. These children prefer starchy and fatty foods, simple carbohydrates, snacks, and processed foods over fruits, vegetables, and proteins (meat, fish, or eggs) [137,138]. It was also shown that a carbohydrate-rich diet correlates positively with the abundance of gut *Candida* [139], while diets with high levels of protein correlate negatively with the abundance of *Candida* in healthy volunteers [140]. Carbohydrates, including glucose and mannose, had the largest impact on *C. albicans* growth and significantly increased the growth of yeast by more than 1000% in comparison with controls [77]. Furthermore, mannitol, sorbitol, xylose, adonitol, and xylitol also significantly increased the growth of *C. albicans* (100–200%) compared with vehicle control. Other metabolites including raffinose, arabinose, trehalose, lactose, galactinol, galactitol, and arabitol had a little or marginal impact on *C. albicans* growth [77]. Therefore, carbohydrates reduction is one of the main elements of anti-candida diets andbecame very popular over the last few decades. An anti-candida diet involves reducing or avoiding added sugars (including honey, jam, and candy), highly-refined carbohydrates (especially any products made with flour), red and cured meats, and dairy products [141]. These diets were suggested for their potential benefits to ASD children, but to date, strong empirical evidence of their effect on *Candida* growth and gut health is lacking. There is insufficient information about their efficacy to make recommendations for their use. No clinical trials to date examined these treatments for ASD.

### 5.2. Probiotic Intervention against Candida Overgrowth

Processed sugars and antibiotics lead to an imbalance in intestinal microbes as well as an increase in the growth of yeast. Therefore, the use of probiotics was suggested as a treatment for ASD children to reduce GI disturbances and the overgrowth of *Candida* [142,143,144]. Indeed, probiotics may improve behavioral symptoms in children with ASD [72]. Unfortunately, only two studies focused on determining the effect of probiotics on the growth of *Candida* sp. in the GI tract. The prospective study was conducted for up to 12 months on preterm newborns (249 objects) treated with *Lactobacillus reuteri* (ATCC 55730; fivedrops daily; 1 × 10^8^ CFU) or *Lactobacillus rhamnosus* (ATCC 53103; one capsule daily; 6 × 10^9^ CFU), and reported the reduction in *Candida* in stools, as well as lower GI symptoms after *L. reuteri* administration [142]. Moreover, infants treated with probiotics showed a statistically significant lower incidence of abnormal neurological outcomes than the control group. Unfortunately, the number of invasive *Candida* infections between the control and probiotic groups was not statistically different. The use of probiotics appears to be effective in the prevention of GI colonization by *Candida*, but not necessarily in the protection from mycotic infections. The oral treatment with *Lactobacillus acidophilus* (strain Rosell-11, 5 × 10^9^ CFU) for 2 months (twice daily) reduced *Candida* colonization in the intestines of 22 children with ASD (45% of participants were on a “sugar-free diet”) [72]. The probiotic supplementation led to a significant decrease in D-arabinitol and the ratio of D-/L-arabinitol in the urine of children with autism, as well as a significant improvement in the ability of ASD children to concentrate and carry out orders [72]. However, while the level of concentration ability and carrying out of orders, was improved in autistic children, the response to emotions was not varied. This study provides only suggestive, not conclusive, evidence regarding the efficacy of probiotics on GI and behavioral symptoms among ASD patients. There is insufficient information regarding the efficacy to make recommendations for their use.

The therapeutic potentials of probiotics in ASD disorder remains controversial [143]. It was shown that some *Lactobacillus* sp. produce a small molecule(1-acetyl-β-carboline, 1-ethoxycarbonyl-β-carboline) that blocks the *C. albicans* yeast-to-filament transition and biofilm formation, an important virulence trait through Yak1, a DYRK1-family kinase [144]. The study byMurzyn et al. [145] documented the effect of capric acid produced by *S. boulardii* in inducing adhesion, yeast-to-hyphae transition, and biofilm formation. Probiotics may manipulate intestinal microbial communities and suppress the growth of pathogens by inducing the host’s production of β-defensin and IgA. Probiotics may be able to fortify the intestinal barrier by maintaining tight junctions and inducing mucin production. Probiotic-mediated immunomodulation may occur through the mediation of cytokine secretion through signaling pathways such as NFκB (nuclear factor kappa-light-chain-enhancer of activated B cells) and MAPK (mitogen-activated protein kinase), which can also affect proliferation and differentiation of immune cells (such as T cells) or epithelial cells. Gut motility may be modulated through the regulation of pain receptor expression and the secretion of neurotransmitters [146].

### 5.3. Antifungal Agents against Candida Overgrowth

The detection of high levels of *Candida* during diagnosis generally requires specific anti-*Candida* treatment. Nystatin and/or fluconazole are the most often used to restore the proper balance of microbiota or to treat *Candida* overgrowth in the intestines of children with ASD [61]. It was shown that all *C. albicans* isolated from the stoolsofchildren with ASD were sensitive to nystatin, fluconazole, and voriconazole, while non-albicans *Candida* showed different sensitivity patterns to the tested antifungals [57]. These results indicate that nystatin, fluconazole, or voriconazole cannot be used as empiric treatment for children with *Candida* overgrowth, but instead a culture of stool samples followed by identification and sensitivity testing should be mandatory for the treatment of ASD patients. Children with ASD after oral nystatin treatment at a dose of 100,000 units, 4 times a day for 70 days improved significantly with regard to the childhood autism rating scale, an indicator of the severity of autism [147]. The concentration of several urine markers for *Candida* growth in the intestine (5-hydroxymethyl 2-furoic acid, furan-2,5-dicarboxylic acid, and arabinose) of autistic children decreased after antifungal treatment [147]. Moreover, the parents and teachers of autistic children observed a decreased hyperactivity, increased eye contact and vocalization, better sleep patterns and concentration, increased imaginative play, reduced stereotypical behaviors such as spinning objects, and better academic performance. Unfortunately, nystatin was never proven to be safe when taken for months or years, and no studies support its use in the treatment of autistic behaviors [148]. Some research demonstrates that combination therapy with antimycotic agents and probiotics showed efficacy in the prevention of GI colonization by *Candida* sp. and in reducing GI symptoms [142]. Infants with *Candida* infections, who treated with antimycotic agents (liposomal Amphotericin B at the initial dose of 1 mgkg^−1^ per day, with a gradual increase up to a maximum of 6 mgkg^−1^ per day, 7 days) and probiotic supplementation (*L. reuteri* or *L. rhamnosus*),experienced fast clinical improvements, with fewer days of treatment in both probiotic groups compared with the control group [142].

On the other hand, antibiotic-treatment-induced gut metabolome and microbiome alterations increase the susceptibility to *C. albicans* colonization and morphogenesis in the GI tract [77]. The alterations at the level of gut metabolites resulting from antibiotics treatment correlate with the increased growth and hyphae formation of *C. albicans* in the GI tract [68,77]. Moreover, fungal species, for example *Saccharomyces boulardii*, *C. albicans*, and *S. cerevisiae* may secrete molecules, such as farnesol, fusel alcohols, tyrosol, and fatty acids, which are autoregulatory molecules for the growth of yeast [149]. These molecules enable fungal cells to regulate adhesion, yeast-to-hyphae transition, and biofilm formation themselves, which in turn facilitate the colonization, invasion, and dissemination of the host. Therefore, understanding numerous signals that regulate *C. albicans* growth and morphogenesis would provide an insight into the balance between commensalism and invasive fungal infection in the GI tract [77].

### 5.4. Fecal Microbiota Transplantation (FMT) and Microbiota Transfer Therapy (MTT) against Candida Overgrowth

The potential use of FMT and itsmodified protocol, MTT, is considered a promising therapy in the treatment of microbiota dysbiosis and GI disorders in ASD children [150,151]. Some research showed that commensal bacteriain the intestine of adult mice, especially the *Bacteroidetes* and *Firmicutes* species, are major resisters for *C. albicans* colonization [152], while *Escherichia coli* super-infection promotes *C. albicans* virulence under certain circumstances [153]. Therefore, it seems that modulating the gut microbiome could alleviate GI symptoms and ASD-related behaviors, but the efficacy of FMT and MTT in ASD is poorly understood and questionable. Only three studies describe the effects of FMT and MTT on children with autism [151,154,155]. An open-label, randomized waitlist-controlled trial confirmed the efficacy and safety of FMT for patients with ASD [154]. The application of FMT (fresh fecal suspension from one anonymous healthy donor), via colonoscopy and gastroscopy under anesthesia three times every 3 months, statistically improved behavioral ASD symptoms (CARS) and shifted the microbiome of ASD patients to a healthy state [154]. MTT-modifiedFMT therapy consisting of 2-week vancomycin treatment followed by a bowel cleanse, and then high dose FMT for 1–2 days and 7–8 weeks of daily maintenance doses, as well as a stomach-acid suppressantadministered for 10weeks to children with ASD, showed an 80% reduction in GI symptoms and a slow but steady improvement in core ASD symptoms [155]. The 2 years after MTT treatment stopped, the authors of the publication verified the long-term effects of these therapies on ASD children [151]. Significant improvements both in GI and behavior symptoms since the end of treatment were noted. Changes in gut microbiota persisted for two years, including overall community diversity and relative abundances of *Bifidobacteria* and *Prevotella*. Unfortunately, none of them described the effect of these therapies on *Candida* overgrowth in the GI tract and a possible improvement in the behavior of children with autism. Only one study showed a decrease in *Candida* and a positive outcome on intestinal inflammation post-FMT in meliorated ulcerative colitis [156]. While FMT appears to be beneficial for patients with meliorated ulcerative colitis, it is not known whether it would also work for the treatment of autistic children with *Candida* overgrowth.

Furthermore, the FDA’s 2020 statement about FMT warns against the use of the colon microbiota transplant procedure due to serious infections caused by multidrug-resistant organisms from the fecal suspension of donors (FDA In Brief: FDA warns about the potential risk of serious infections caused by multidrug-resistant organisms related to the investigational use of Fecal Microbiota for Transplantation). Additionally, due to the possibility of transmission of infectious diseases with transplanted material (e.g., implantation of cancer cells of colon carcinoma [157] or other cancerogenic factors, such as HPV virus [158]), thescreening for pathogens and other risks should be performed before the FMT and MTT procedure. However, these few studies do not provide information about the fecal material characteristic, its donors, methods of its preparation, and standardization. Therefore, treating with FMT and MTT can be quite risky. At this moment, in clinical practice, FMT was only used in the treatment of recurrent *Clostridium difficile* (rCDIs) [159] and restricted to patients not responding positively to standard treatment procedures [160]. Clinical trials to increase the understanding of the benefits of FMT and TMM in autism are still needed.

## 6. Conclusions

It is important to realize that the etiology of autism is still unknown. Although, some interventions may be effective in alleviating some symptoms and improving skills that may help autistic persons lead more productive lives, so far, people suffering from ASD do not have access to an effective cure or a completely effective therapy. Therefore, new scientific discoveries regarding the etiology of ASD and the potential treatment options for this disorder are enthusiastically received by people with autism and their care providers. *Candida* overgrowth in the intestines is suggested to be one of many possible causes of autism. Unfortunately, the few studies available do not lead to clear conclusions. The presence of *Candida* sp. in stool samples from children with ASD and healthy controls in most studies are not significant (6/9), or no data are available (2/9) (Table 1). No significant differences between groups do not allow a confirmation that *Candida* overgrowth refers to children with autism or that it may cause autism. The size of the groups (test vs. control) is too small (ranging from 1 to 58 ASD children), which probably affects the statistical insignificance of the results. Moreover, the results of most studies do not fully support the hypothesis that *Candida* overgrowth is correlated with GI problems and affects ASD behavioral symptoms. Furthermore, it would seem that modulatingthe microbiota composition in the gut may decrease *Candida* overgrowth, help reduce GI problems and autism symptoms, but evidence from human studies suggesting beneficial effects of a sugar-free diet, probiotic supplementation, FMT and MTT treatment in ASD is limited and inconclusive. Current data should not encourage the use of these modalities in the therapy of ASD, and making such changes to the nutrition of autistic children could have a potentially negative impact on their development. Surely, furtherdetailed and better-designed clinical studies are required to elucidate the possible involvement of *Candida* in the pathophysiology of ASD.

## Figures and Tables

**Table 1:** *Candida* sp. in stool samples from children with ASD.

Study Groups	*Candida* sp. Identified in a Stool Samples	Statistical Significance (ASD vs. Control)	Impact on Children with ASD	Ref.
- 35 children with ASD (32M/3F) - 37 neurotypical children (28M/9F)	*Candida*	Not statistically significant	- Not significantly correlated with GI scores;	[55]
			- Significantly higher ATEC scores;	
			- Effect on ASD behavioral symptoms.	
- 1 male with ASD	*C. parapsilosis*	No data	- Significant increase in eye contact and use of spontaneous language, decrease in self-stimulatory behavior shortly after beginning antifungal therapy (100,000 Units nystatin 4 times a day + alternating weeks of Nizoral or Diflucan (2 mg/kg)) and gluten- and casein-free diet for 5 months; - Increased learning speed in the schooling program, increased verbal labeling, and increased spontaneous verbal initiations;	[56]
			- Decreased CARS score rating from 43 (severely autistic) to a value of 29 (nonautistic).	
- 50 children with ASD - 36 children controls (brother/sister) - 50 healthy children	*C. glabrata* *C. parapsilosis*	Not statistically significant	- Presence of *Candida* sp. did not affect the severity of symptoms in ASD children;	[57]
	*C. tropicalis*		- No significant differences in GI symptoms.	
	*C. albicans*			
	*C. krusei*			
- 28 children with ASD (22M/6F) - 25 neurotypical children	*Candida*	Not statistically significant	- No significant differences in GI symptoms;	[58]
			- Lack of associations between GI microflora population levels and autism severity.	
- 40 children with ASD (31M/9F) - 40 neurotypical children (28M/12F)	*Candida*	Partially significant	- An expansion of *Candida* in the gut mycobiota of autistic individuals may negatively impact GI abnormalities.	[46]
- 47 children with ASD (40M/7F) - 33 healthy children (24M/9F)	*Candida*	Statistically significant	- Presence of *Candida* spp.in 57.5% of ASDs (27/47) and none in the control group; - *C. albicans* was the most frequently isolated (16 times/27 total cases) yeast;	[59]
			- Identification of aggressive form (pseudohyphae) of *Candida* spp.;	
			- Increased counts of *Candida* spp. was observed even in the absence of GI symptoms;	
			- Low–mild gut inflammation and augmented intestinal permeability were confirmed;	
			- Inflammation correlated to disease severity (CARS) and intestinal permeability impairment was related to GI symptom type.	
- 58 children with ASD - 39 healthy typical children	*Candida*	Not statistically significant	- No differences of yeast infection among stools from ASDs and healthy controls; - The strong correlation of GI symptoms with autism severity indicates that children with more severe autism are likely to have more severe GI symptoms and vice versa.	[60]
- 1 child	*C*. *albicans* *C.krusei* *C. glabrata*	No data	- *C. albicans* growth in intestines may cause lower absorption of carbohydrates and minerals, and higher toxin levels, which are thought to contribute to autistic behaviors.	[61]
- 33 children with ASD - 16 healthy children	*Candida*	Not statistically significant	- No significant differences between groups; - The results of study do not fully support the hypothesis that the composition of the GI microbiota or significantly altered ratios of these microbes change susceptibility to ASD development in children.	[62]

Legends: M—male;F—female;ATEC—Autism Treatment Evaluation Checklist; CARS—Childhood Autism Rating Scale; GI—gastrointestinal.

## Data Availability

Not applicable.

## References

[1] Riglin L., Wootton R.E., Thapar A., Livingston L., Langley K., Collishaw S., Tagg J., Smith G.D., Stergiakouli E., Tilling K.M. (2021). Variable emergence of autism spectrum disorder symptoms from childhood to early adulthood. Am. J. Psychiatry.

[2] Patterson P.H. (2011). Maternal infection and immune involvement in autism. Trends Mol. Med..

[3] Zerbo O., Qian Y., Yoshida C., Grether J.K., Van de Water J., Croen L.A. (2015). Maternal infection during pregnancy and autism spectrum disorders. J. Autism Dev. Disord..

[4] Chen S., Zhao S., Dalman C., Karlsson H., Gardner R. (2021). Association of maternal diabetes with neurodevelopmental disorders: Autism spectrum disorders, attention-deficit/hyperactivity disorder and intellectual disability. Int. J. Epidemiol..

[5] Amsyari F., Mukarromah N. (2003). Reconsideration of early childhood vaccination (an epidemiological study on relationships between vaccination and autism). Folia Med. Indones..

[6] Blaylock R.L. (2008). The danger of excessive vaccination during brain development. Med. Veritas.

[7] Skogheim T.S., Weyde K.V.F., Engel S.M., Aase H., Surén P., Øie M.G., Biele G., Reichborn-Kjennerud T., Caspersen I.H., Horning M. (2021). Metal and essential element concentrations during pregnancy and associations with autism spectrum disorder and attention-deficit/hyperactivity disorder in children. Environ. Int..

[8] Kaur I., Behl T., Aleya L., Rahman M.H., Kumar A., Arora S., Akter R. (2021). Role of metallic pollutants in neurodegeneration: Effects of aluminium, lead, mercury, and arsenic in mediating brain impairment events and autism spectrum disorder. Environ. Sci. Pollut. Res..

[9] Rosen N.J., Yoshida C.K., Croen L.A. (2007). Infection in the first 2 years of life and autism spectrum disorders. Pediatrics.

[10] Gondalia S.V., Palombo E.A., Knowles S.R., Austin D.W. (2010). Gastrointestinal microbiology in autistic spectrum disorder: A review. Rev. Med. Microbiol..

[11] Bransfield R.C. (2009). Preventable cases of autism: Relationship between chronic infectious diseases and neurological outcome. Pediatric Health.

[12] Kovtun A.S., Averina O.V., Alekseeva M.G., Danilenko V.N. (2020). Antibiotic resistance genes in the gut microbiota of children with autistic spectrum disorder as possible predictors of the disease. Microb. Drug Resist..

[13] Champagne-Jorgensen K., Kunze W.A., Forsythe P., Bienenstock J., McVey Neufeld K.A. (2019). Antibiotics and the nervous system: More than just the microbes?. Brain Behav. Immun..

[14] Nitschke A., Deonandan R., Konkle A.T. (2020). The link between autism spectrum disorder and gut microbiota: A scoping review. Autism.

[15] Srikantha P., Mohajeri M.H. (2019). The possible role of the microbiota-gut-brain-axis in autism spectrum disorder. Int. J. Mol. Sci..

[16] Settanni C.R., Bibbò S., Ianiro G., Rinninella E., Cintoni M., Mele M.C., Cammarota G., Gasbarrini A. (2021). Gastrointestinal involvement of autism spectrum disorder: Focus on gut microbiota. Exp. Rev. Gastroenterol. Hepatol..

[17] Lasheras I., Seral P., Latorre E., Barroso E., Gracia-García P., Santabárbara J. (2020). Microbiota and gut-brain axis dysfunction in autism spectrum disorder: Evidence for functional gastrointestinal disorders. Asian J. Psychiatry.

[18] De Magistris L., Familiari V., Pascotto A., Sapone A., Frolli A., Iardino P., Carteni M., De Rosa M., Francavilla R., Riegler G. (2010). Alterations of the intestinal barrier in patients with autism spectrum disorders and in their first-degree relatives. J. Pediatr. Gastroenterol.Nutr..

[19] Muthuirulan P. (2016). Leaky Gut Syndrome: Mystery Illness Triggered by Candida albicans. J. Nutr. Health Food Eng..

[20] Jyonouchi H. (2009). Food allergy and autism spectrum disorders: Is there a link?. Curr. Allergy Asthma. Rep..

[21] Jyonouchi H. (2010). Autism spectrum disorders and allergy: Observation from a pediatric allergy/immunology clinic. Exp. Rev. Clin. Immunol..

[22] Ashwood P., Krakowiak P., Hertz-Picciotto I., Hansen R., Pessah I., Van de Water J. (2011). Elevated plasma cytokines in autism spectrum disorders provide evidence of immune dysfunction and are associated with impaired behavioral outcome. Brain Behav. Immun..

[23] Cristiano C., Lama A., Lembo F., Mollica M.P., Calignano A., MattaceRaso G. (2018). Interplay between peripheral and central inflammation in autism spectrum disorders: Possible nutritional and therapeutic strategies. Front. Physiol..

[24] Jyonouchi H., Geng L. (2019). Associations between monocyte and T cell cytokine profiles in autism spectrum disorders: Effects of dysregulated innate immune responses on adaptive responses to recall antigens in a subset of ASD children. Int. J. Mol. Sci..

[25] Matta S.M., Hill-Yardin E.L., Crack P.J. (2019). The influence of neuroinflammation in autism spectrum disorder. Brain Behav. Immun..

[26] Siniscalco D., Schultz S., Brigida A.L., Antonucci N. (2018). Inflammation and neuro-immune dysregulations in autism spectrum disorders. Pharmaceuticals.

[27] Doernberg E., Hollander E. (2016). Neurodevelopmental disorders (ASD and ADHD): DSM-5, ICD-10, and ICD-11. CNS Spectr..

[28] El-Ansary A. (2020). GABA and glutamate imbalance in autism and their reversal as novel hypothesis for effective treatment strategy. Autism Dev. Disord..

[29] Márquez-Caraveo M.E., Ibarra-González I., Rodríguez-Valentín R., Ramírez-García M.Á., Pérez-Barrón V., Lazcano-Ponce E., Vela-Amieva M. (2021). Brief report: Delayed diagnosis of treatable inborn errors of metabolism in children with autism and other neurodevelopmental disorders. J. Autism Dev. Disord..

[30] Garcia-Gutierrez E., Narbad A., Rodríguez J.M. (2020). Autism spectrum disorder associated with gut microbiota at immune, metabolomic, and neuroactive level. Front. Neurosci..

[31] Guissart C., Latypova X., Rollier P., Khan T.N., Stamberger H., McWalter K., Cho M.T., Kjaergaard S., Weckhuysen S., Lesca G. (2018). Dual molecular effects of dominant RORA mutations cause two variants of syndromic intellectual disability with either autism or cerebellar ataxia. Am. J. Hum. Genet..

[32] Melke J. (2008). Autism: Which genes are involved. Clin. Neuropsychiat..

[33] Williams E.L., Casanova M.F., Switala A.E., Li H., Qiu M. (2013). Transposable elements occur more frequently in autism-risk genes: Implications for the role of genomic instability in autism. Translat. Neurosci..

[34] Risch N., Hoffmann T.J., Anderson M., Croen L.A., Grether J.K., Windham G.C. (2014). Familial recurrence of autism spectrum disorder: Evaluating genetic and environmental contributions. Am. J. Psychiatry.

[35] Eshraghi R.S., Deth R.C., Mittal R., Aranke M., Kay S.I.S., Moshiree B., Eshraghi A.A. (2018). Early disruption of the microbiome leading to decreased antioxidant capacity and epigenetic changes: Implications for the rise in autism. Front. Cell Neurosci..

[36] Loomes R., Hull L., Mandy W.P.L. (2017). What is the male-to-female ratio in autism spectrum disorder? A systematic review and meta-analysis. J. Am. Acad. Child Adolesc. Psych..

[37] El-Ansary A., Bhat R.S., Zayed N. (2020). Gut microbiome and sex bias in autism spectrum disorders. Curr. Behav. Neurosci. Rep..

[38] Dhaliwal K.K., Orsso C.E., Richard C., Haqq A.M., Zwaigenbaum L. (2019). Risk factors for unhealthy weight gain and obesity among children with autism spectrum disorder. Int. J. Mol. Sci..

[39] Noguera M. (2020). Human gut microbiome metabolism and autism spectrum disorder. J. Agric. Life Sci..

[40] McElhanon B.O., McCracken C., Karpen S., Sharp W.G. (2014). Gastrointestinal symptoms in autism spectrum disorder: A meta-analysis. Pediatrics.

[41] Gondalia S.V., Palombo E.A., Knowles S.R., Cox S.B., Meyer D., Austin D.W. (2012). Molecular characterization of gastrointestinal microbiota of children with autism (with and without gastrointestinal dysfunction) and their neurotypical siblings. Autism Res..

[42] Fujimori S. (2015). What are the effects of proton pump inhibitors on the small intestine?. World J. Gastroenterol..

[43] Flint H.J. (2012). The impact of nutrition on the human microbiome. Nutr. Rev..

[44] Parracho H.M., Bingham M.O., Gibson G.R., McCartney A.L. (2005). Differences between gut microflora of children with autistic spectrum disorders and that of healthy children. J. Med. Microbiol..

[45] Williams B.L., Hornig M., Buie T., Bauman M.L., Cho Paik M., Wick I., Bennett A., Jabado O., Hirschberg D.L., Lipkin W.I. (2011). Impaired carbohydrate digestion and transport and mucosal dysbiosis in the intestines of children with autism and gastrointestinal disturbances. PLoS ONE.

[46] Strati F., Cavalieri D., Albanese D., De Felice C., Donati C., Hayek J., Jousson O., Leoncini S., Renzi D., Calabro A. (2017). New evidences on the altered gut microbiota in autism spectrum disorders. Microbiome.

[47] Finegold S.M., Dowd S.E., Gontcharova V., Liu C., Henley K.E., Wolcott R.D., Youn E., Summanen P.H., Granpeesheh D., Dixon D. (2010). Pyrosequencing study of fecal microflora of autistic and control children. Anaerobe.

[48] Finegold S.M. (2011). Desulfovibrio species are potentially important in regressive autism. Med. Hypotheses.

[49] Plaza-Díaz J., Gómez-Fernández A., Chueca N., Torre-Aguilar M.J.D.L., Gil Á., Perez-Navero J.L., Flores-Rojas K., Martin-Borreguero P., Solis-Urra P., Ruiz-Ojeda F.J. (2019). Autism spectrum disorder (ASD) with and without mental regression is associated with changes in the fecal microbiota. Nutrients.

[50] Chin V.K., Yong V.C., Chong P.P., Amin Nordin S., Basir R., Abdullah M. (2020). Mycobiome in the gut: A multiperspective review. Med. Inflamm..

[51] Erdogan A., Rao S.S. (2015). Small intestinal fungal overgrowth. Curr. Gastroenterol. Rep..

[52] Zou R., Wang Y., Duan M., Guo M., Zhang Q., Zheng H. (2021). Dysbiosis of gut fungal microbiota in children with autism spectrum disorders. J. Autism Dev. Disord..

[53] Tiew P.Y., Mac Aogain M., Ali N.A.T.B.M., Thang K.X., Goh K., Lau K.J., Chotirmall S.H. (2020). The mycobiome in health and disease: Emerging concepts, methodologies and challenges. Mycopathologia.

[54] Pappas P.G., Rex J.H., Sobel J.D., Filler S.G., Dismukes W.E., Walsh T.J., Edwards J.E. (2004). Guidelines for treatment of candidiasis. Clin. Inf. Dis..

[55] Patel J., Kang D.W., Adams J., Krajmalnik-Brown R. (2018). Analysis of Yeast and Fungi in Children with ASD vs. Neurotypical Controls. Undergraduate Thesis.

[56] Shaw W., Baptist J., Geenens D. (2010). Immunodeficiency, gastrointestinal Candidiasis, wheat and dairy sensitivity, abnormal urine arabinose, and autism: A case study. N. Am. J. Med. Sci..

[57] Ahmed S.A.S., Meheissen M.A., Azouz H.G., Ashry M.H., Roshdy Y.S., Gad H.A., Ibrahim A.E. (2017). Study of Candida species in stool of children with autism spectrum disorders in Alexandria, Egypt. Microbiol. Res. J. Int..

[58] Gondalia S.V., Palombo E.A., Knowles S.R., Austin D.W. (2010). Faecal microbiota of individuals with autism spectrum disorder. E-J. Appl. Psychol. Clin. Soc. Issues.

[59] Iovene M.R., Bombace F., Maresca R., Sapone A., Iardino P., Picardi A., Marotta R., Schiraldi C., Siniscalco D., Serra N. (2017). Intestinal dysbiosis and yeast isolation in stool of subjects with autism spectrum disorders. Mycopathologia.

[60] Adams J.B., Johansen L.J., Powell L.D., Quig D., Rubin R.A. (2011). Gastrointestinal flora and gastrointestinal status in children with autism–comparisons to typical children and correlation with autism severity. BMC Gastroenterol..

[61] Kantarcioglu A.S., Kiraz N., Aydin A. (2016). Microbiota–gut–brain axis: Yeast species isolated from stool samples of children with suspected or diagnosed autism spectrum disorders and in vitro susceptibility against nystatin and fluconazole. Mycopathologia.

[62] Jendraszak M., Gałęcka M., Kotwicka M., Regdos A., Pazgrat-Patan M., Andrusiewicz M. (2021). Could selected gut microorganisms be diagnostic biomarkers for autism spectrum disorders? Study based on a commercial microbiota test. Res. Square.

[63] Emam A.M., Mamdouh E., Abdelrahim S. (2012). *Candida albicans* infection in autism. J. Am. Sci..

[64] El-Shouny W.A., Ismail S., Elzawawy N., Hegazy S. (2016). Efficacy of herbal control of the yeasts isolated from autistic children. GJBAHS.

[65] Lo H.J., Kohler J.R., DiDomenico B., Loebenberg D., Cacciapuoti A., Fink G.R. (1997). Nonfilamentous *C. albicans* mutants are avirulent. Cell.

[66] Carlisle P.L., Banerjee M., Lazzell A., Monteagudo C., López-Ribot J.L., Kadosh D. (2009). Expression levels of a filament-specific transcriptional regulator are sufficient to determine *Candida albicans* morphology and virulence. Proc. Natl. Acad. Sci. USA.

[67] Toenjes K.A., Munsee S.M., Ibrahim A.S., Jeffrey R., Edwards J.E., Johnson D.I. (2005). Small-molecule inhibitors of the budded-to-hyphal-form transition in the pathogenic yeast *Candida albicans*. Antimicrob. Agents Chemother..

[68] Bar-Yosef H., Vivanco Gonzalez N., Ben-Aroya S., Kron S.J. (2017). Chemical inhibitors of *Candida albicans* hyphal morphogenesis target endocytosis. Sci. Rep..

[69] Colombo A.L., Padovan A.C.B., Chaves G.M. (2011). Current knowledge of *Trichosporon* spp. and *Trichosporonosis*. Clin. Microbiol. Rev..

[70] Laswi I., Shafiq A., Al-Ali D., Burney Z., Pillai K., Salameh M., Mhaimeed N., Zakaria D., Chaari A., Yousri N.A. (2021). A comparative pilot study of bacterial and fungal dysbiosis in neurodevelopmental disorders and gastrointestinal disorders: Commonalities, specificities and correlations with lifestyle. Microorganisms.

[71] Shaw W., Kassen E., Chaves E. (1995). Increased urinary excretion of analogs of Krebs cycle metabolites and arabinose in two brothers with autistic features. Clin. Chem..

[72] Kałużna-Czaplińska J., Błaszczyk S. (2012). The level of arabinitol in autistic children after probiotic therapy. Nutrients.

[73] Noto A., Fanos V., Barberini L., Grapov D., Fattuoni C., Zaffanello M., Casanova A., Fenu G., De Giacomo A., De Angelis M. (2014). The urinary metabolomics profile of an Italian autistic children population and their unaffected siblings. J.Matern.-Fetal Neonatal Med..

[74] Lord R.S., Burdette C.h.K., Bralley J.A. (2004). Urinary markers of yeast overgrowth. Integ. Med..

[75] Becker J., Boles E. (2003). A modified *Saccharomyces cerevisiae* strain that consumes L-Arabinose and produces ethanol. Appl. Environ. Microbiol..

[76] Richard P., Verho R., Putkonen M., Londesborough J., Penttila M. (2003). Production of ethanol from L-arabinose by Saccharomyces cerevisiae containing a fungal L-arabinose pathway. FEM Yeast Res..

[77] Gutierrez D., Weinstock A., Antharam V.C., Gu H., Jasbi P., Shi X., Dirks B., Krajmalnik-Brown R., Maldonado J., Guinan J. (2020). Antibiotic-induced gut metabolome and microbiome alterations increase the susceptibility to *Candida albicans* colonization in the gastrointestinal tract. FEMS Microbiol. Ecol..

[78] Lord R.S. (2003). Urinary markers of intestinal yeast. Townsend Lett. Dr. Patients.

[79] Fonseca A. (1992). Utilization of tartaric acid and related compounds by yeasts: Taxonomic implications. Can. J. Microbiol..

[80] Fonseca A., Fell J.W., Kurtzman C.P., Spencer-Martins I. (2000). *Candida tartarivorans* sp. nov., an anamorphic ascomycetous yeast with the capacity to degrade L(+)- and meso-tartaric acid. Int. J. Syst.Evol. Microbiol..

[81] Markova N. (2019). Dysbiotic microbiota in autistic children and their mothers: Persistence of fungal and bacterial wall-deficient L-form variants in blood. Sci. Rep..

[82] Hughes H.K., Ashwood P. (2018). Anti-*Candida albicans* IgG antibodies in children with autism spectrum disorders. Front. Psychiatry.

[83] Janeway C.J., Travers P., Walport M. (2001). The Distribution and Functions of Immunoglobulin Isotypes. Immunobiology: The Immune System in Health and Disease.

[84] Rabalais G.P., Samiec T.D., Bryant K.K., Lewis J. (1996). Invasive candidiasis in infants weighing more than 2500 grams at birth admitted to a neonatal intensive care unit. Pediatr. Infect. Dis. J..

[85] Rimland B. (1988). Candida-caused autism. Autism Res. Rev.Inern..

[86] Crook W.G. (1984). Yeast can affect behavior and learning. Acad.Ther..

[87] Carter C.J. (2019). Autism genes and the leukocyte transcriptome in autistic toddlers relate to pathogen interactomes, infection and the immune system. A role for excess neurotrophic sAPPα and reduced antimicrobial Aβ. Neurochem. Int..

[88] Colina A.R., Aumont F., Deslauriers N., Belhumeur P., de Repentigny L. (1996). Evidence for degradation of gastrointestinal mucin by *Candida albicans* secretory aspartyl proteinase. Inf. Immun..

[89] Noverr M.C., Huffnagle G.B. (2004). Regulation of *Candida albicans* morphogenesis by fatty acid metabolites. Infect. Immun..

[90] García C., Tebbji F., DaigneaultMLiu N.N., Kohler J.R., Allen-Vercoe E., Sellam A. (2017). The human gut microbial metabolome modulates fungal growth via the TOR signalling pathway. mSphere.

[91] Iliev I.D., Leonardi I. (2017). Fungal dysbiosis: Immunity and interactions at mucosal barriers. Nat. Rev. Immunol..

[92] Kumamoto C.A. (2011). Inflammation and gastrointestinal *Candida* colonization. Curr. Opin. Microbiol..

[93] Wheeler M.L., Limon J.J., Underhill D.M. (2017). Immunity to commensal fungi: Detente and disease. Annu. Rev. Pathol..

[94] Roth S., Ruland J. (2013). Caspase recruitment domain-containing protein 9 signalling in innate immunity and inflammation. Trends Immunol..

[95] Czakai K., Leonhardt I., Dix A., Bonin M., Linde J., Einsele H., Kurzai O., Loeffler J. (2016). Kruppel-like Factor 4 modulates interleukin-6 release in human dendritic cells after in vitro stimulation with *Aspergillus fumigatus* and *Candida albicans*. Sci. Rep..

[96] Conti H.R., Gaffen S.L. (2015). IL-17-mediated immunity to the opportunistic fungal pathogen *Candida albicans*. J. Immunol..

[97] Moorlag S., Roring R.J., Joosten L.A.B., Netea M.G. (2018). The role of the interleukin-1 family in trained immunity. Immunol. Rev..

[98] Russo A.J., Krigsman A., Jepson B., Wakefield A. (2009). Low serum myeloperoxidase in autistic children with gastrointestinal disease. Clin. Exp. Gastroenterol..

[99] Lehrer R.I., Cline M.J. (1969). Leukocyte myeloperoxidase deficiency and disseminated candidiasis: The role of myeloperoxidase in resistance to *Candida* infection. J. Clin.Investig..

[100] Lanza F. (1998). Clinical manifestation of myeloperoxidase deficiency. J. Mol. Med..

[101] Ceylan M.F., Hesapcioglu S.T., Yavas C.P., Senat A., Erel O. (2012). Serum ischemia-modified albumin levels, myeloperoxidase activity and peripheral blood mononuclear cells in autism spectrum disorder (ASD). J. Autism Dev. Disord..

[102] Topal Z., Tufan A.E., Karadag M., Gokcen C., Akkaya C., Sarp A.S., Bahsi I., Kilinc M. (2021). Evaluation of peripheral inflammatory markers, serum B12, folate, ferritin levels and clinical correlations in children with autism spectrum disorder (ASD) and attention deficit hyperactivity disorder (ADHD). Nordic. J. Psychiatry.

[103] Aratani Y. (2018). Myeloperoxidase: Its role for host defense, inflammation, and neutrophil function. Arch. Biochem. Biophys..

[104] Stanzani M., Orciuolo E., Lewis R., Kontoyiannis D.P., Martins S.L., St John L.S., Komanduri K.V. (2005). *Aspergillus fumigatus* suppresses the human cellular immune response via gliotoxin-mediated apoptosis of monocytes. Blood.

[105] Hubmann R., Hilgarth M., SchnablSPonath E., Reiter M., Demirtas D., Sieghart W., Valent P., Zielinski Ch Jager U., Shehata M. (2013). Gliotoxin is a potent NOTCH 2 transactivation inhibitor and efficiently induces apoptosis in chronic lymphocytic leukaemia (CLL) cells. Br. J. Haematol..

[106] Shah D., Larsen B. (1991). Clinical isolates of yeast produce a gliotoxin-like substance. Mycopathologia.

[107] Podzorski R., Herron M., Fast D., Nelson R. (1989). Pathogenesis of candidiasis immunosuppression by cell wall mannan catabolites. Arch. Surg..

[108] Witkin S.S. (1985). Defective immune responses in patients with recurrent candidiasis. Infect. Med..

[109] Ho J., Yang X., Nikou S.A., Kichik N., Donkin A., Ponde N.O., Richardson J.P., Gratacap R.L., Archambault L.S., Zwirner C.P. (2019). Candidalysin activates innate epithelial immune responses via epidermal growth factor receptor. Nat. Commun..

[110] Naglik J.R., Gaffen S.L., Hube B. (2019). Candidalysin: Discovery and function in *Candida albicans* infections. Curr. Opin. Microbiol..

[111] Hornby J.M., Jensen E.C., Lisec A.D., Tasto J.J., Jahnke B., Shoemaker R., Dussault P., Nickerson K.W. (2001). Quorum sensing in the dimorphic fungus *Candida albicans* is mediated by farnesol. Appl. Environ. Microbiol..

[112] Kasper L., Konig A., Koenig P.A., Gresnigt M.S., Westman J., Drummond R.A., Lionakis M.S., Gross O., Ruland J., Naglik J.R. (2018). The fungal peptide toxin Candidalysin activates the NLRP3 inflammasome and causes cytolysis in mononuclear phagocytes. Nat. Commun..

[113] Rogiers O., Frising U.C., Kucharikova S., Jabra-Rizk M.A., van Loo G., Van Dijck P., Wullaert A. (2019). Candidalysin crucially contributes to Nlrp3 inflammasome activation by *Candida albicans* hyphae. MBio.

[114] Leonhardt I., Spielberg S., Weber M., Albrecht-Eckardt D., Bläss M., Claus R., Barz D., Scherlach K., Hertweck C., Löffler J. (2015). The fungal quorum-sensing molecule farnesol activates innate immune cells but suppresses cellular adaptive immunity. mBio.

[115] Noverr M.C., Phare S.M., Toews G.B., Coffey M.J., Huffnagle G.B. (2001). Pathogenic yeasts *Cryptococcus neoformans* and *Candida albicans* produce immunomodulatory prostaglandins. Infect. Immun..

[116] Kim Y.G., Udayanga K.G., Totsuka N., Weinberg J.B., Nunez G., Shibuya A. (2014). Gut dysbiosis promotes M2 macrophage polarization and allergic airway inflammation via fungi-induced PGE(2). Cell Host Microbe.

[117] Tan T.G., Lim Y.S., Tan A., Leong R., Pavelka N. (2019). Fungal symbionts produce prostaglandin E2 to promote their intestinal colonization. Front. Cell Infect. Microbiol..

[118] Burrus C.J. (2012). A biochemical rationale for the interaction between gastrointestinal yeast and autism. Med. Hypothesis.

[119] Denaro F.J., López-Ribot J.L., Chaffin W.L. (1995). Adhesion of *Candida albicans* to brain tissue of *Macaca mulatta* in an ex vivo assay. Infect. Immun..

[120] Jong A.Y., Stins M.F., Huang S.H., Chen S.H.M., Kim K.S. (2001). Traversal of *Candida albicans* across human blood-brain barrier in vitro. Infect Immun.

[121] Navarathna D.H., Munasinghe J., Lizak M.J., Nayak D., McGavern D.B., Roberts D.D. (2013). MRI confirms loss of blood–brain barrier integrity in a mouse model of disseminated candidiasis. NMR Biomed..

[122] Ophelders D.R., Gussenhoven R., Lammens M., Küsters B., Kemp M.W., Newnham J.P., Payne M.S., Kallapur S.G., Jobe A.H., Zimmermann L.J. (2016). Neuroinflammation and structural injury of the fetal ovine brain following intra-amniotic *Candida albicans* exposure. J. Neuroinflamm..

[123] Masi A., Quintana D.S., Glozier N., Lloyd A.R., Hickie I.B., Guastella A.J. (2015). Cytokine aberrations in autism spectrum disorder: A systematic review and meta-analysis. Mol. Psychiatry.

[124] Herman A.P., Bochenek J., Król K., Krawczyńska A., Antushevich H., Pawlina B., Herman A., Romanowicz K., Tomaszewska-Zaremba D. (2016). Central Interleukin-1β suppresses the nocturnal secretion of melatonin. Med. Inflamm..

[125] Skelly D.T., Griffin E.W., Murray C.L., Harney S., O’Boyle C., Hennessy E., Dansereau M.A., Nazmi A., Tortorelli L., Rawlins J.N. (2019). Acute transient cognitive dysfunction and acute brain injury induced by systemic inflammation occur by dissociable IL-1-dependent mechanisms. Mol. Psychiatry.

[126] Griffiths J.S., Camilli G., Kotowicz N.K., Ho J., Richardson J.P., Naglik J.R. (2021). Role for IL-1 family cytokines in fungal infections. Front. Microbiol..

[127] Reichelt K., Knivsberg A. (2009). The possibility and probability of a gut-to-brain connection in autism. Am. Clin. Psychiatry.

[128] Dhossche D., Applegate H., Abraham A., Maertens P., Bland L., Bencsath A., Martinez J. (2002). Elevated plasma gamma-aminobutyric acid (GABA) levels in autistic youngsters: Stimulus for a GABA hypothesis of autism. Med. Sci. Monitor.

[129] Fatemi S.H., Reutiman T.J., Folsom T.D., Thuras P.D. (2009). GABA A receptor downregulation in brains of subjects with autism. J. Autism Dev. Disord..

[130] Muller C.L., Anacker A.M., Veenstra-Vander Weele J. (2016). The serotonin system in autism spectrum disorder: From biomarker to animal models. Neuroscience.

[131] Connors S.L., Matteson K.J., Sega G.A., Lozzio C.B., Carroll R.C., Zimmerman A.W. (2006). Plasma serotonin in autism. Pediatr. Neurol..

[132] Cook E.H., Leventhal B.L. (1996). The serotonin system in autism. Curr. Opin. Pediatr..

[133] Marler S., Ferguson B.J., Lee E.B., Peters B., Williams K.C., McDonnell E., Macklin E.A., Levitt P., Gillespie C.H., Anderson G.M. (2016). Brief report: Whole blood serotonin levels and gastrointestinal symptoms in autism spectrum disorder. J. Autism Dev. Disord..

[134] Doenyas C. (2018). Dietary interventions for autism spectrum disorder: New perspectives from the gut-brain axis. Physiol. Behav..

[135] Yang J., Fu X., Liao X., Li Y. (2020). Effects of gut microbial-based treatments on gut microbiota, behavioral symptoms, and gastrointestinal symptoms in children with autism spectrum disorder: A systematic review. Psychiatry Res..

[136] Volkert V.M., Vaz P.C.M. (2010). Recent studies on feeding problems in children with autism. J. Appl.Behav. Anal..

[137] Sharp W.G., Berry R.C., McCracken C., Nuhu N.N., Marvel E., Saulnier C.A., Klin A., Jones W., Jaquess D.L. (2013). Feeding problems and nutrient intake in children with autism spectrum disorders: A meta-analysis and comprehensive review of the literature. J. Autism Dev. Disord..

[138] Vissoker R.E., Latzer Y., Gal E. (2015). Eating and feeding problems and gastrointestinal dysfunction in Autism Spectrum Disorders. Res. Autism Spectr. Disord..

[139] Lam S., Zuo T., Ho M., Chan F.K.L., Chan P.K.S., Ng S.C. (2019). Review article: Fungal alterations in inflammatory bowel diseases. Aliment. Pharmacol. Ther..

[140] Pan C., Hoffmann C., Dollive S., Grunberg S., Chen J., Li H., Wu G.D., Lewis J.D., Bushman F.D. (2013). Archaea and fungi of the human gut microbiome: Correlations with diet and bacterial residents. PloS ONE.

[141] Jacobs G., Kjaer J. (2012). Beat Candida through Diet: A Complete Dietary Programme for Suffers of Candidiasis.

[142] Romeo M.G., Romeo D.M., Trovato L., Oliveri S., Palermo F., Cota F., Betta P. (2011). Role of probiotics in the prevention of the enteric colonization by *Candida* in preterm newborns: Incidence of late-onset sepsis and neurological outcome. J. Perinatol..

[143] Liu J., Wan G.B., Huang M.S., Agyapong G., Zou T.L., Zhang X.Y., Liu Y.W., Song Y.Q., Tsai Y.C., Kong X.J. (2019). Probiotic therapy for treating behavioral and gastrointestinal symptoms in autism spectrum disorder: A systematic review of clinical trials. Curr. Med. Sci..

[144] MacAlpine J., Daniel-Ivad M., Liu Z., Yano J., Revie N.M., Todd R.T., Stogios P.J., Sanchez H., O’Meara T.R., Tompkins T.A. (2021). A small molecule produced by *Lactobacillus* species blocks *Candida albicans* filamentation by inhibiting a DYRK1-family kinase. Nat. Commun..

[145] Murzyn A., Krasowska A., Stefanowicz P., Dziadkowiec D., Łukaszewicz M. (2010). Capric acid secreted by *S. boulardii* inhibits *C. albicans* filamentous growth, adhesion and biofilm formation. PLoS ONE.

[146] Hemarajata P., Versalovic J. (2013). Effects of probiotics on gut microbiota: Mechanisms of intestinal immunomodulation and neuromodulation. Ther. Adv. Gastroenterol..

[147] Shaw W., Kassen E., Chaves E. (2000). Assessment of antifungal drug therapy in autism by measurement of suspected microbial metabolites in urine with gas chromatography-mass spectrometry. Clin. Pract. Altern. Med..

[148] Zimmerman B., Weber E. (1985). *Candida* and “20th-century disease”. CMAJ Can. Med. Assoc. J..

[149] Shareck J., Belhumeur P. (2011). Modulation of morphogenesis in *Candida albicans* by various small molecules. Eukaryotic Cell.

[150] Tan Q., Orsso C.E., Deehan E.C., Kung J.Y., Tun H.M., Wine E., Madsen K.L., Zwaigenbaum L., Haqq A.M. (2021). Probiotics, prebiotics, synbiotics, and fecal microbiota transplantation in the treatment of behavioral symptoms of autism spectrum disorder: A systematic review. Autism Res..

[151] Kang D.W., Adams J.B., Coleman D.M., Pollard E.L., Maldonado J., McDonough-Means S., Caporaso J.G., Krajmalnik-Brown R. (2019). Long-term benefit of microbiota transfer therapy on autism symptoms and gut microbiota. Sci. Rep..

[152] Fan D., Coughlin L.A., Neubauer M.M., Kim J., Kim M.S., Zhan X., Simms-Waldrip T.R., Xie Y., Hooper L.V., Koh A.Y. (2015). Activation of HIF-1alpha and LL-37 by commensal bacteria inhibits *Candida albicans* colonization. Nat. Med..

[153] Akagawa G., Abe S., Yamaguchi H. (1995). Mortality of *Candida albicans*-infected mice is facilitated by superinfection of *Escherichia coli* or administration of its lipopolysaccharide. J. Infect. Dis..

[154] Zhao H., Gao X., Xi L., Shi Y., Peng L., Wang C., Zou L., Yang Y. (2019). Mo1667 fecal microbiota transplantation for children with an autism spectrum disorder. Gastrointest. Endoscop..

[155] Kang D.W., Adams J.B., Gregory A.C., Borody T., Chittick L., Fasano A., Khoruts A., Geis E., Maldonado J., McDonough-Means S. (2017). Microbiota transfer therapy alters gut ecosystem and improves gastrointestinal and autism symptoms: An open-label study. Microbiome.

[156] Leonardi I., Paramsothy S., Doron I., Semon A., Kaakoush N.O., Clemente J.C., Faith J.J., Borody T.J., Mitchell H.M., Colombel J.F. (2020). Fungal trans-kingdom dynamics linked to responsiveness to fecal microbiota transplantation (FMT) therapy in ulcerative colitis. Cell Host Microbe.

[157] Mullish B.H., Quraishi M.N., Segal J.P., McCune V.L., Baxter M., Marsden G.L., Moore D.J., Colville A., Bhala N., Iqbal T.H. (2018). The use of faecal microbiota transplant as treatment for recurrent or refractory *Clostridium difficile* infection and other potential indications: Joint British Society of Gastroenterology (BSG) and Healthcare Infection Society (HIS) guidelines. Gut.

[158] Cheng Y.W., Phelps E., Ganapini V., Khan N., Ouyang F., Xu H., Khanna S., Tariq R., Friedman-Moraco R.J., Woodworth M.H. (2019). Fecal microbiota transplantation for the treatment of recurrent and severe *Clostridium difficile* infection in solid organ transplant recipients: A multicenter experience. Am. J. Transplant..

[159] McDonald L.C., Gerding D.N., Johnson S., Bakken J.S., Carroll K.C., Coffin S.E., Dubberke E.R., Garey K.W., Gould C.V., Kelly C. (2018). Clinical practice guidelines for *Clostridium difficile* infection in adults and children: 2017 update by the Infectious Diseases Society of America (IDSA) and Society for Healthcare Epidemiology of America (SHEA). Clin. Infect. Dis..

[160] Tan X., Johnson S. (2019). Fecal microbiota transplantation (FMT) for *C. difficile* infection, just say ‘No’. Anaerobe.

